# Characteristics of a Population with Gender Incongruence Assisted at a Specialized Outpatient Service in the City of Ribeirão Preto

**DOI:** 10.1055/s-0042-1742407

**Published:** 2022-02-09

**Authors:** Sérgio Henrique Pires Okano, Silvio Antônio Franceschini, Maria Rita Lerri, Omero Benedicto Poli-Neto, Luiz Gustavo Oliveira Brito, Lúcia Alves da Silva Lara

**Affiliations:** 1 Human Reproduction Division, Department of Gynecology and Obstetrics, Faculdade de Medicina de Ribeirão Preto, Universidade de São Paulo, Ribeirão Preto, SP, Brazil; 2 Department of Tocogynecology, Faculdade de Ciências Medicas, Universidade Estadual de Campinas, Campinas, SP, Brazil

**Keywords:** transgender people, gender dysphoria, sexual and gender minorities, pessoas transgêneras, disforia de gênero, minorias sexuais e de gênero

## Abstract

**Objective** To identify the age when individuals first perceive gender incongruence (GI) and to compare sociodemographic data of female-to-male (FtM) and male-to-female (MtF) transgender individuals assisted at an outpatient service.

**Methods** The present cross-sectional study was conducted through a review of the medical records of individuals diagnosed with GI at a single specialized outpatient service in the city of Ribeirão Preto, state of São Paulo, Brazil.

**Results** A total of 193 medical records from 2010 to 2018 were evaluated, and 109 (56.5%) patients had GI since childhood. The FtM transgender individuals perceived GI in childhood more often than the MtF transgender individuals (odds ratio [OR]: 2.06, 95% confidence interval [95%CI]: 1.11–3.81) Unattended hormone use was highest among the MtF group (69.6% versus 32.3%; OR: 4.78, 95%CI: 2.53–9.03). All of the individuals who were engaged in prostitution or were diagnosed with a sexually-transmitted infection, including HIV, were in the MtF group.

**Conclusion** Despite the more prevalent perception of GI in childhood among the FtM group, social issues were more prevalent among the MtF group, which may be the result of social marginalization.

## Resumo

**Objetivo** Identificar o período da vida em que indivíduos indentificaram pela primeira vez sua incongruência de gênero (IG), e comparar os dados sociodemográficos de homens e mulheres transgêneros (trans) atendidos em um ambulatório.

**Métodos** Estudo transversal realizado por meio de revisão dos prontuários de pessoas com IG em ambulatório especializado de Ribeirão Preto, São Paulo, Brasil.

**Resultados** Foram avaliados 193 prontuários de 2010 a 2018, e 109 (56.5%) pacientes apresentavam IG desde a infância. Homens trans perceberam a IG na infância com mais frequência do que as mulheres trans (razão de probabilidades [RP]: 2.06, intervalo de confiança de 95% [IC95%]: 1.11–3.81). O uso de hormônio sem supervisão foi maior entre as mulheres trans (69.6% *versus* 32.3%; RP: 4.78; IC95%: 2.53–9.03). Todos as pessoas que estavam inseridas na prostituição ou que apresentavam algum diagnóstico de infecção sexualmente transmissível, incluindo o HIV, eram mulheres trans.

**Conclusão** Apesar da percepção mais prevalente da IG na infância entre homens trans, os agravos sociais foram mais prevalentes entre as mulheres trans, o que pode ser resultado da marginalização social.

## Introduction

Gender identity is usually established when an individual begins to develop the physical characteristics that are typical of a mature male or female. Gender incongruence (GI) is defined by a difference between a person's gender identity and the sex assigned at birth. A meta-analysis 1 showed that the overall prevalence rate of GI was of 4.6 per 100 thousand individuals, with 6.8 transgender (trans) women (male-to-female, MtF) per 100 thousand individuals, and 2.6 trans men (female-to-male, FtM) per 100 thousand individuals.

Little is known about the complex processes that underlie the formation of gender identity, but interactions between psychosocial and biological factors appear to be important. 2 A child's perception of being a female or male arises gradually, and about 6% of boys and 12% of girls resist the socially-imposed stereotypes regarding clothing and games and play, and engage in behaviors considered inappropriate for their biological sex. 2 Research 3 on gender typification (GT) has indicated that this occurs early in childhood, although children vary significantly in the degree to which they become typified. Prospective data has suggested that 2% to 27% of children who have characteristics of GI will maintain this condition in adulthood, and that the presence of GI in adulthood is associated with a stronger expression of GI in childhood. 4 However, children and adolescents who experience GI need the attention of health care providers, because GI can cause psychological distress and the desire to alter the body's appearance. 5

Adequate care for children and adolescents who have GI can improve their mental health and well-being. This population has high rates of anxiety, depression, and suicidal tendencies, 6 7 possibly because of discrimination, stigmatization, 8 9 10 and rejection by their families and society in general. 6 11

Therefore, the identification of the age when an individual perceives his or her gender identity is important because this may facilitate the development of protocols that promote their health and well-being. The present study aimed to identify the age when individuals first perceive GI.

## Methods

The present is a cross-sectional study in which data were obtained through a review of the medical records at a multidisciplinary service in Brazil (Gender Incongruence Outpatient Clinic, Faculdade de of Medicina de Ribeirão Preto, Universidade de São Paulo [FMRP-USP], São Paulo, Brazil) by a team (composed of a psychiatrist, a psychologist, a gynecologist, and a speech therapist) that is devoted to providing comprehensive health care for the transgender population. All medical and psychological records from July 2010 to July 2018 were examined, and the records of individuals who had a first appointment for hormone therapy only or hormone therapy with surgery were reviewed. Subjects under 18 years of age and those whose medical records did not contain the necessary data for the proposed analyses were excluded.

At the first visit, each patient was welcomed by a nurse who explained the care to be offered. The patient subsequently visited with a gynecologist, who assessed the clinical data through a semi-structured interview. A complete physical examination was performed, and the patient was referred to the psychologist for an evaluation and follow-up. During the follow-up, screening tests for breast, bowel, prostate, and cervical cancers are requested, following the guidelines for specific screening in the transgender population. 12 13 14 15 16 17 18 19 20 21 22 23 24 In addition, test pertaining to endocrine-metabolic screening, such as lipidogram, glycemia, thyroid-stimulating hormone (TSH), and bone densitometry are also requested, according to the protocols suggested by the Brazilian Ministry of Health. 24 The patients undergoing hormonal treatment were submitted to collections of testosterone, estradiol, hematocrit, liver enzymes, and prolactin for dose adjustment and risk assessment. 21 These assessments are crucial, since this population faces barriers to access health care in other services. As depression and anxiety disorders are prevalent among transgender individuals, 15 16 skilled professionals and mental health specialists perform the evaluation and provide care for this people in our service. Transgender individuals attending the clinic also receive guidance about prevention of sexually-transmitted infections (STIs), the benefits of the use of Preexposure prophylaxis (PrEP) for HIV, contraception, and cessation of harmful habits such as smoking, and drug and alcohol absuse. Adolescents and children diagnosed with GI are assisted in a private environment, and support is also provided for their families.

Sociodemographic variables, including age and employment status, were collected from the registration form. The presence of any disease related to cardiovascular risks, such as diabetes mellitus, hypertension, coronary artery disease, dyslipidemia, and newly-identified thrombosis, were self-reported. The history of STIs was recorded, and laboratory results were obtained from the electronic records. Habits that are harmful to health, such as smoking, drinking alcohol, and the use of illicit drugs, were also recorded. To assess the age at the perception of GI, the semi-structured questionnaire asked the question: “In what period of your lifetime did you realize you felt like a woman (or man)?”. The possible answers were “childhood” (age < 10 years), “adolescence” (age 10 to 20 years), or “adulthood” (> 20 years). This stratification follows the periods of life established by the World Health Organization (WHO). 12

For the quantitative variables, an exploratory data analysis was performed considering the measures of central position and dispersion. For the qualitative variables, absolute and relative frequencies were presented. The Chi-squared test was used to verify which qualitative variables were significantly associated with the group variable (FtM or MtF). The statistical analyses were performed using the SAS (SAS Isntitute, Cary, NC, United States) software, version 9.4.

The present research project was submitted for review to the Ethics in Research Committee of FMRP-USP (CAAE: 94406518.0.0000.5440). The practical activities of this project were guided by the ethical norms of Resolution 466/2012 of the Brazilian National Health Council on Research with Human Beings. The present study was based on a retrospective review of medical records, so informed consent was not required. The authors have no conflicts of interest to declare.

## Results

**Fig. 1:**
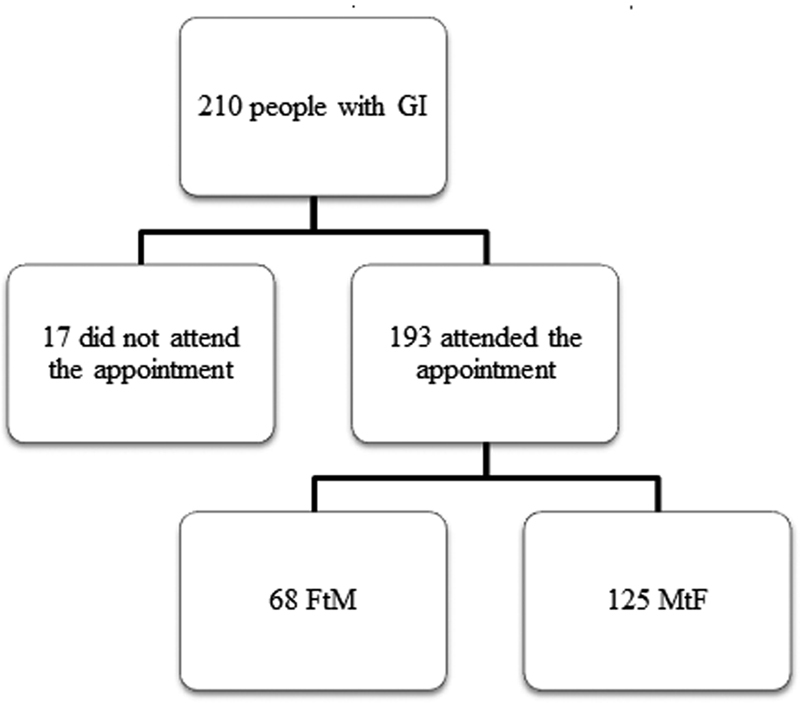
Enrollment of individuals with gender incongruence.

There were 210 individuals potentially eligible for inclusion ( Figure 1 ). We excluded 17 patients because they did not attend their appointments and enrolled the remaining 193 individuals ( Figure 1 ).

A total of 125 (64.8%) subjects composed the MtF group (mean age: 27 years; standard deviation [SD]: 9.0 years) and 68 (35.2%) composed the FtM group (mean age: 27.2 years; SD: 9.2 years). A comparison of the sociodemographic characteristics of the two groups ( Table 1 ) indicated that a significantly larger number of people in the MtFs group were undergoing hormone therapy (69.6% versus 32.3% for the FtM group; *p* < 0.01). The rates of prostitution, STIs, and HIV positivity were significantly higher in the MtF than in the FtM group (odds ratio [OR]: 2.06; 95% confidence interval [95%CI]: 1.11–3.81).

**Table 1:** Sociodemographic characteristics of the study participants

Variables	Total n (%)	Male-to-Female n (%)	Female-to-Male n (%)	*p* -value
	193 (100.0)	125 (64.8)	68 (35.2)	−
*Age (years)*				
< 18	17 (8.8)	11 (8.8)	6 (8.8)	−
18 to 29	129 (66.8)	75 (60.0)	54 (79.4)	−
30 to 50	43 (22.3)	36 (28.8)	7 (10.3)	−
> 50	4 (2.1)	3 (2.4)	1 (1.5)	−
*Employment status*				0.11
Employed	128 (66.3)	88 (70.4)	40 (58.8)	−
Student	36 (18.7)	18 (14.4)	18 (26.5)	−
Unemployed	29 (15.0)	19 (15.2)	10 (14.7)	−
*Clinical risk factors*				
Anxiety/depression	19 (9.8)	11 (8.8)	8 (11.8)	0.51
Smoking	50 (35.9)	27 (21.6)	23 (33.8)	0.06
Alcohol use	35 (18.1)	22 (17.6)	13 (19.1)	0.79
Drug addiction	23 (11.9)	17 (13.6)	8 (11.7)	0.71
Previous hormone use	109 (56.5)	87 (69.6)	22 (32.3)	< 0.01

**Fig. 2:**
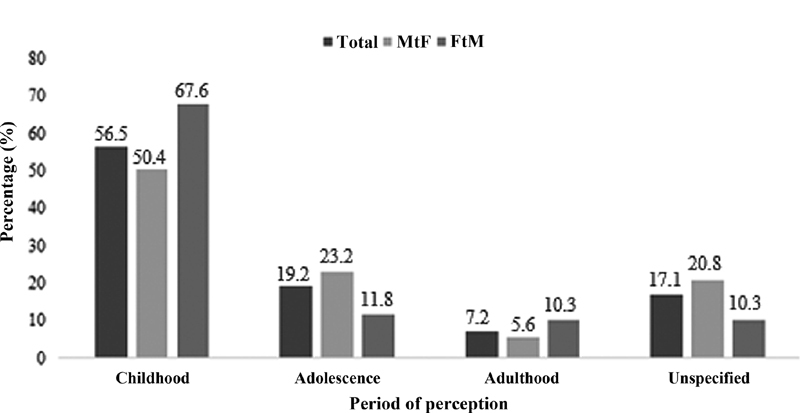
Period in life (in percentage) in which the perception of GI occurred by gender (n = 193).

The first perception of GI occurred in childhood for 56.5% of the subjects, during adolescence for 19.2% of the subjects, and during adulthood for 7.2% of the subjects ( Figure 2 ). Among the MtF participants, 63 (50.4%) reported the first perception of GI during childhood, 29 (23.2%), during adolescence, and 7 (5.6%), during adulthood; among the FtM participants, 46 (67.6%) reported the first perception of GI during childhood, 8 (11.8%), during adolescence, and 7 (10.3%), during adulthood. The Chi-squared test indicated a higher percentage of individuals in the FtM group who first perceived GI during childhood (OR: 2.06; 95%CI: 1.11–3.81).

All subjects who reported engaging in prostitution (4.1%), who had STIs (13.5%), or who were HIV-positive (11.4%) were in the MtF group. The overall prevalence of smoking was of 35.9%, that of use of alcohol was of 18.1%, and that of use of psychoactive substances was of 11.9%. More MtF than FtM participants reported hormonal self-medication prior to the first appointment (69.6% versus 32.3% respectively; OR: 4.78; 95%CI: 2.53–9.03). Individuals in the MtF group who reported the perception of GI in childhood had a higher prevalence of HIV positivity than those who reported perception during adolescence and adulthood ( *p* = 0.04).

## Discussion

The present study aimed to identify the ages of individuals when they first perceived GI. Most of our subjects perceived GI during childhood, followed by adolescence and adulthood. These data are important for the development of interventions that may help families to better accommodate the free expression of gender identity by their children, and may also help to improve the outcomes of gender dysphoria treatment in adolescents. Additionally, the use of family-based interventions may improve the understanding and compassion towards individuals with GI, and help to prevent family rejection, a major problem among this population. 6 The development of protocols to assist children who are expressing the characteristics of GI is challenging due to the lack of studies on the impact of familial, social, and school rejection upon these children. Schools can be hostile environments for people with GI, and there are reports of prejudice, violence, and abuse in learning institutions, the major factors that restrict access to education and lead to greater marginalization of individuals with GI. 13 Although almost 20% of our study subjects reported being students, the FtM participants were more likely to be students than those in the MtF group.

Aside from providing recognition of GI, health care services provide few coping strategies for individuals with GI, particularly for those who present at young ages. In our clinic, for example, the vast majority of individuals are adults when they present with GI. Programs aimed at family assistance and care for children with GI may help reduce problems such as marginalization and infection by HIV and other STIs, and may also reduce unhealthy behaviors, such as the use of alcohol, illicit drugs, tobacco, and self-medication with hormones. Family reception and relationship may help protect against depression in this group. 6 Previous prospective studies 4 have shown that only 2% to 27% of children who manifested GI during childhood maintained this condition during adolescence and adulthood. Therefore, when a child reaches adolescence and the GI has persisted, there is a high probability that it will remain during adulthood. 14 In the present study, we did not examine the childhood behaviors of our subjects that could suggest the presence of GI.

The prevalence of depression symptoms in our population was low, of only 9.8% for the whole sample (8.8% in MtF group and 11.8% in the FtM group). Conversely, a European study 15 reported that the prevalence of depression among a population of individuals with GI was of 38% during their study period (2007 to 2010). Our lower rate of depression symptoms may be due to our multidisciplinary approach, which was specifically designed to assist our transgender population. We plan to further examine this issue in future studies. We believe it is important to better train medical teams to consider complaints related to depression and anxiety and to evaluate emotional relationships and the need for emotional support in individuals with GI. Suicidal ideation and attempted suicide due to mental health problems are up to 20 times more common in individuals with GI than in the general population. 16

Two-thirds of our sample reported being employed at the time of presentation to our service. The prevalence of prostitution in our sample was extremely low (4.2%), and all of these individuals were in the MtF group. Transgender women (MtF individuals) often experience social exclusion and discrimination, and these lead to reduced access to employment; consequently, they engage in sex work to earn income. In Brazil, 90% of MtF individuals resort to prostitution as a source of income at some point in their lives. 17 In addition, all of our subjects who were positive for HIV and other STIs were in the MtF group, which is in line with previous reports. 17 A Brazilian study 19 showed that the prevalence of HIV positivity in MtF individuals was 17.6%, 5 times higher than in the general population, and that the HIV positivity reached 25% in some regions.

In our sample, one-third of the MtF participants and one-quarter of the FtM participants reported the use of tobacco. This corroborates data from a previous study 20 on smoking by transgender people in the United States. The higher rate of smoking by individuals with GI is an especially serious public health concern, because it increases the risk factor for thrombosis, especially for MtF individuals taking estrogen. 21

The prevalence of alcohol consumption in our sample was of 18.1%. Kerr-Corrêa et al. 22 evaluated 304 MtF individuals, and found that three-quarters of them consumed alcohol. This is also a public health concern because alcohol consumption can lower inhibitions and increase the risk of engaging in unprotected sex and using illicit drugs.

The overall prevalence of the use of illicit substances (marijuana, cocaine, and ecstasy) by our subjects was of 11.9% (13.6% among the MtF group and 11.7% among the FtM group). According to Day et al., 23 the prevalence of use of psychoactive substances is 2.5 to 4 times higher among transgender than cisgender people. Day et al. 23 have also reported that the higher prevalence of use of psychoactive drugs by individuals with GI may be a consequence of their social marginalization and the increased risk of psychological problems.

Many patients with GI first present to our service as adults who were already using hormone medications. In general, the limited access to health care institutions, mistaken preconceptions regarding GI by some health care professionals, and fear of discrimination prevent transgender people from initiating adequate hormone therapy. 24 However, the indiscriminate use of hormones without guidance from health care providers can lead to serious side effects, such as thromboembolic disease, breast and endometrial cancers, erythrocytosis, coronary and cerebrovascular diseases, liver dysfunction, and transient infertility. 21

It is important to provide guidance to help health care professionals welcome this population in their healthcare services. Perception of GI at a young age is very common, so family education and childcare programs may help reduce the marginalization of these people, which often results from family rejection. 6 Marginalization is associated with depression, anxiety, the use of illicit drugs, and risky behaviors, 23 which, in turn, increase the risk for acquiring STIs. 22

A limitation of the present study is that our data were obtained through a retrospective review of reports provided by caregivers of individuals who presented with GI. Therefore, there is a possibility of detection, memory, and/or diagnostic bias, because some subjects may not have reported certain sensitive information. One strength of the present study is that all the data were obtained by an interdisciplinary group of professionals who have experience in assisting individuals with GI, and gynecology residents are part of this team that provides health care to this population. The American College of Obstetricians and Gynecologists argued that obstetrician-gynecologists (Ob-Gyns) should provide care for transgender people. 25 Therefore, we think that the findings of the present study – which wereobtained in a gynecological setting –may contribute to the development of specific protocols that provide more integrated health care to individuals with GI.

## Conclusion

Most individuals with GI first perceive the onset of this condition during childhood, and a higher percentage of FtM individuals than MtF individuals first perceive GI during this period of life. Regarding the sample of the present study, there were no cases of prostitution nor STIs among the FtM group. The MtF and FtM groups were similar in that they had high prevalences of smoking and use of illicit drugs and alcohol, but the MtF group was much more likely to self-medicate with hormones.
